# Can drones save lives and money? An economic evaluation of airborne delivery of automated external defibrillators

**DOI:** 10.1007/s10198-022-01531-0

**Published:** 2022-10-30

**Authors:** Johann W. A. Röper, Katharina Fischer, Mina Carolina Baumgarten, Karl Christian Thies, Klaus Hahnenkamp, Steffen Fleßa

**Affiliations:** 1 Department of General Business Administration and Health Care Management, Faculty of Law and Economics, University of Greifswald, Friedrich-Loeffler-Straße 70, 17489 Greifswald, Germany; 2 Department of Anesthesiology and Intensive Care Medicine, University of Greifswald, Ferdinand-Sauerbruch-Straße, 17489 Greifswald, Germany; 3 Department of Anesthesiology and Critical Care, EvKB, Bielefeld University Hospitals, Burgsteig 13, 33617 Bielefeld, Germany

**Keywords:** AED, Out of hospital cardiac arrest, Drone, Emergency medical care, UAS

## Abstract

**Background:**

Out-of-hospital cardiac arrest is one of the most frequent causes of death in Europe. Emergency medical services often struggle to reach the patient in time, particularly in rural areas. To improve outcome, early defibrillation is required which significantly increases neurologically intact survival. Consequently, many countries place Automated External Defibrillators (AED) in accessible public locations. However, these stationary devices are frequently not available out of hours or too far away in emergencies. An innovative approach to mustering AED is the use of unmanned aerial systems (UAS), which deliver the device to the scene.

**Methods:**

This paper evaluates the economic implications of stationary AED versus airborne delivery using scenario-based cost analysis. As an example, we focus on the rural district of Vorpommern-Greifswald in Germany. Formulae are developed to calculate the cost of stationary and airborne AED networks. Scenarios include different catchment areas, delivery times and unit costs.

**Results:**

UAS-based delivery of AEDs is more cost-efficient than maintaining traditional stationary networks. The results show that equipping cardiac arrest hot spots in the district of Vorpommern-Greifswald with airborne AEDs with a response time < 4 min is an effective method to decrease the time to the first defibrillation The district of Vorpommern-Greifswald would require 45 airborne AEDs resulting in annual costs of at least 1,451,160 €.

**Conclusion:**

In rural areas, implementing an UAS-based AED system is both more effective and cost-efficient than the conventional stationary solution. When regarding urban areas and hot spots of OHCA, complementing the airborne network with stationary AEDs is advisable.

**Supplementary Information:**

The online version contains supplementary material available at 10.1007/s10198-022-01531-0.

## Introduction

Out-of-hospital cardiac arrest (OHCA) is one of the most frequent causes of death in developed countries. In 2019, Germany recorded an incidence of 118.5 OHCA patients per 100,000 inhabitants [1, 2]. A comparison to other European countries shows that much lower mortality rates with the more favourable patient outcome can be achieved, given the right measures are taken [3]. This strongly points to a need for action and an assessment of alternatives for German emergency medical services (EMS).

OHCA occurs when the pumping function of the heart suddenly stops and blood circulation ceases. As a result, vital organs, e. g. the brain, are no longer supplied with oxygen and loose viability [4]. Therefore, immediate treatment of OHCA is critical to achieve higher chances of a positive outcome for the patient. To ensure oxygen supply, it is important to perform cardiopulmonary resuscitation (CPR) as quickly as possible. In case of shockable rhythms, defibrillation can stimulate the natural heart function. Therefore, automatic external defibrillators (AED) can help in improving OHCA outcome [5], if applied promptly.

In urban areas, the probability of survival of an OHCA is higher than in rural areas. On the one hand, the probability of OHCA being witnessed is higher in urban regions. On the other hand, EMS response times are shorter. In rural areas with low population density the EMS response time are much longer; the low population density in rural areas does not allow for a higher number of EMS stations. Furthermore, terrain (e.g. mountains, rivers or lakes) challenges access speed, as rural traffic infrastructure is often less developed compared to urban regions. This calls for innovative solutions in pre-hospital care for rural areas [6–8].

Over the last decades, a number of approaches have been implemented to reduce the therapy-free interval and the time to the first defibrillation in OHCA. Amongst these are telephone or video-assisted Cardiopulmonary Resuscitation (CPR) [9–11], Community-First-Responder (CFR)[12] concepts as well as extensive networks of publicly accessible AEDs. For example, Denmark, Sweden, and the Netherlands have already installed stationary AED networks to achieve short defibrillation intervals [13].In Germany, however, existing AEDs are often neither strategically distributed, nor available 24/7 [14].

Stationary AED networks face several functional challenges. Most people who witness cardiac arrest do not know the location of the nearest AED and have to pick it up on foot. Consequently, the access time to the device can be quite long and binds first-responding capacities, which may be needed to perform CPR. The accessibility problem can be relaxed to a certain degree by digitizing and mapping the networks [15, 16]. Still, accessibility often depends on the opening hours of the site where devices are placed. Because AEDs are frequently stolen or damaged, they are kept in safe places, e.g. in the office of facility managers. However, opening hours limit the AED´s accessibility to first-responders, further reducing the stationary AED´s benefits in OHCA situations. Increasing the number of stationary AEDs does not necessarily solve the problem of access outside office hours.

An innovative approach to overcome the disadvantages of stationary AED networks could be their mobilization with the help of modern technology. This requires a device that could deliver the AED with high speed to any place on demand 24 h a day at a cost lower than that of existing airborne solutions, such as helicopters [17]. Unmanned aircraft systems (UAS), are a possible way to deliver AEDs to the site of a cardiac arrest and not having first responders retrieve them in the first place [18]. It has been shown that UAS networks for AED provision can significantly reduce the time to first defibrillation [19, 20].

Besides the proven medical benefits of timely AED delivery by UAS, operating UAS-networks has also been rated as cost effective. A study published by Bauer et al. [21] rated the primary outcome of operating UAS networks by using the incremental cost-effectiveness-ratio (ICER). The ICER was calculated by the ratio of financial costs to additional life years gained compared to current EMS outcomes. In three scenarios, the coverage of a certain rural area by 80%, 90% and 100% by a UAS network was evaluated, with results showing live-saving potential and cost-effectiveness for any modelled area coverage. ICER ranged between 12.158 € and 23.568 € with life years gained between 1477 and 1933 years in annual average.

The results by Bauer et al. [21] are especially important to evaluate the cost-effectiveness of UAS networks in terms of additional life years gained. However, little is known about the cost-systematic of operating the necessary network of drones. Further, a comparison of the UAS network as the innovation to stationary AED-networks, representing the existing and widely used solution, has not been undertaken as of yet.

In this paper, we would like to close this research gap by comparing the cost-effectiveness of both systems. In the next section, we develop the cost functions for both alternatives. We evaluate their effectiveness by comparing their cost to the AED´s access time. Afterwards, we will present the respective results and discuss the findings. The paper closes with recommendations for the German EMS.

## Methods

### Setting

This study aims to evaluate and improve the efficient supply of AEDs for the response to OHCA. Using a scenario-based approach to cover possible uncertainties, we compared the UAS-supported AED delivery to the common stationary network regarding their cost and efficiency in a rural district.

As time is of outstanding importance in achieving favourable OHCA outcomes, we use the delivery time of AEDs as the main impact factor of an AED system´s efficiency. Using clinical outcomes such as DALYs or QALYs would be preferable, however, they are not available as of yet. Therefore, time is represented as a physical outcome in this analysis as an accepted and widely used [22–24] factor in cost-effectiveness analysis. We calculate the cost-effectiveness by comparing the yearly costs with the respective effectiveness of the alternatives.

The analytical planning period to calculate the cost of operating an AED system is set to one year. This gives us the yearly costs (including annual depreciation acc. to straight-line method) of every scenario. We choose this period to achieve comparability between the introduced scenarios. This time horizon is used as the analytical frame, which we use to develop further assumptions that form our scenario analysis.

Our analysis is based on the research project MV|LIFE|DRONE—Pilot [25] in the district of Vorpommern-Greifswald in the state of Mecklenburg-Vorpommern in North-Eastern Germany. The research group MV|LIFE|DRONE evaluates the feasibility of UAS-based AED delivery since 2019 and works on the implementation of drones to innovate medical transports.

With an area of 3930 km^2^ and a population of some 236,600 (2019), the district of Vorpommern-Greifswald is rural, sparsely populated and peripheral for German standards. Consequently, the results of this analysis can be representative of other rural areas and may also give implications for peri-urban and urban settings.

### Analysis

#### Cost modelling

This analysis assumes a cost function with interval-fixed costs to evaluate the total and unit costs per km^2^ covered by AED services. [26] To determine the size of a provisioned area, which depends on the delivery time of the AED, and the number of AED locations, a Christaller comb pattern is applied [27]. The Christaller comb is a very formal approach, whose practical applicability can be considered critically in principle. However, there is no existing AED system in the region of experience or generally in Germany [14]. Few AEDs are deployed in Germany, their location is neither mapped or systematically structured—we cannot speak of a functional AED network. Therefore, to establish an AED network especially in rural areas, building pads for both mobile and stationary AED from scratch seems feasible and necessary. Our approach is to supply a specific area with AEDs, regardless of the population within. This corresponds to German healthcare practice, which aims to achieve an equal supply of health services in any place. This leads to the premise to operate the same amount of AEDs in a given area.

Every AED´s location has an operational radius that limits the distance which can be covered within a specific response time. To avoid both overlapping and unsupplied areas of provision, the circle´s curves are formed into lines, creating a hexagonal structure around the AED´s location. On adjoining hexagons borders, the distance to either hexagon´s centre, where the AEDs are placed, is exactly the same. Finally, the total costs to provide an AED network covering a given region are calculated by multiplying the number of hexagons, thus the number of necessary AEDs, with the cost per AED unit [26]. The hexagonal approach can be applied to both stationary and airborne AED networks.

Formula (3) calculates the stationary AED´s operational radius *r*_*s*_. The outward and return journeys of a first responder are taken into account, which halves the stationary AED´s operational range. A stationary AED´s operational range is further reduced by detours that have to be walked to retrieve the device. The airborne AED operational radius *r*_*m*_, calculated by (7), only includes the outward journey as retrieval by first responders is not necessary. In contrast, UAS can minimize distances by flying the shortest way by Euclidean distance. However, times for the UAS to take off and land have to be taken into account.

The catchment areas *A*_*s,m*_ for both stationary (2) and airborne AEDs (6) are calculated by radian measure and depend on their operational radii. [28] Total costs result by multiplying the unit costs with the number of AEDs needed to provide sufficient area coverage. The number of AED units is calculated by a trunc function, which gives the next higher integer of the number of AED locations.

Based on experiences made in MV|LIFE|DRONE, we assume a higher technical failure risk associated with using UAS, e.g. due to crashes, technical failures or high winds. Consequently, this model assumes UAS being redundantly provisioned at a rate of 50%. This firstly ensures a high availability and balances possible deficits in technical maturity. Secondly, we assume a very conservative estimation, as data on the availability and reliability of medical UAS is neither available nor researched as of yet. Following the explanations above, the cost functions of stationary AEDs are as follows:

1 $$K_{s} = k_{s} \cdot trunc\left( {\frac{x}{{A_{s} }}} \right)$$

For total costs, with

2 $$A_{s} = 3 \cdot r_{s}^{2} \cdot \sin \left( {60} \right)$$

for one stationary AED´s hexagonal catchment area and

3 $$r_{s} = \frac{{\left( {d - a_{s} } \right) \cdot b_{s} }}{2}$$

for the operational radius.

Total costs for the coverage of a given area are, therefore, calculated as

4 $$K_{s} = k_{s} \cdot trunc \, \left[ {\frac{x}{{3 \cdot \left( {\frac{{\left( {d - a_{s} } \right) \cdot b_{s} }}{2}} \right)^{2} \cdot {\text{sin}}\left( {60} \right)}}} \right]$$

Total costs for airborne AEDs can be calculated as

5 $$K_{m} = k_{m} \cdot trunc\left\{ {\left( {trunc\left[ {\frac{x}{{A_{m} }}} \right]} \right) \cdot 1,5} \right\}$$

for total costs, with

6 $$A_{m} = 3 \cdot r_{m}^{2} \cdot \sin \left( {60} \right)$$

to calculate the UAS´ hexagonal catchment area and

7 $$r_{m} = \left( {d - a_{m} } \right) \cdot b_{m}$$

for the UAS´ operational radius, which determines the size of the hexagon.

Total stationary costs, therefore, are calculated as

8 $$K_{m} = k_{m} \cdot trunc\left\{ {\left( {trunc\left[ {\frac{x}{{3 \cdot \left\{ {\left( {d - a_{m} } \right) \cdot b_{m} } \right\}^{2} \cdot \sin \left( {60} \right)}}} \right]} \right) \cdot 1,5} \right\}$$

The models determinants are Variable: Parameter; ${A}_{m,s}$*:* Catchment area; ${a}_{s}$: time to find the AED; ${a}_{m}$: time to take off and land the UAS; ${b}_{s}$: Speed of a brisk walk; ${b}_{m}$: Speed of a UAS; $d$: response time; ${k}_{s}$: unit costs stationary AED; ${k}_{m}$: unit costs airborne AED; $x$: Area covered; ${K}_{s}$: Total costs area coverage with stationary AEDs; ${K}_{m}$: Total costs area coverage with airborne AEDs.

Fixed costs for either scenario comprise of the acquisition cost of the UAS and the AED. Costs for the UAS-pad as a fully automated ground station are integrated as well as costs for installing a stationary publicly accessible AED. Maintenance cost, which has to be conducted regularly, is regarded as sunk cost and therefore integrated into the fixed costs for both airborne and stationary solutions. Due to the high and still rising degree of automation we experienced in our field project MV|LIFE|DRONE, personnel cost for piloting are not considered. In fact, they will presumably further lose in importance.

Variable costs generally depend on the cost per AED's use. For stationary AEDs, the variable costs consist of the cost of the battery and the electrodes. In the case of airborne AEDs, the costs of charging the UAS´ battery would have to be added. However, considering their probable utilization, the variable costs of using the stationary or airborne AEDs are low. They can be regarded as non-relevant for decision-making and are therefore neglected.

### Sensitivity analysis

In the second step, to illustrate the impact of different unit costs and response times on results, a sensitivity analysis is conducted. By keeping all but one model determinant constant, it gives the highest possible cost of an airborne AED scenario, that equates the cost of the stationary scenario, or vice versa. In this course, the response time *d* and the unit costs ${k}_{s}$ and ${k}_{m}$ of each scenario are regarded.

$$\overline{{K_{s} }} \left( {\overline{{k_{s} }} ,\overline{d},\overline{{a_{s} }} ,\overline{{b_{s} }} ,\overline{x}} \right) = k_{m} \cdot trunc\left\{ {\left( {trunc\left[ {\frac{{\overline{x}}}{{3 \cdot \left\{ {\left( {\overline{d} - \overline{{a_{m} }} } \right) \cdot \overline{{b_{m} }} } \right\}^{2} \cdot \sin \left( {60} \right)}}} \right]} \right) \cdot 1,5} \right\}$$

$$\overline{{K_{m} }} \left( {\overline{{k_{m} }} ,\overline{d},\overline{{a_{m} }} ,\overline{{b_{m} }} ,\overline{x}} \right) = k_{s} \cdot trunc\left[ {\frac{{\overline{x}}}{{3 \cdot \left\{ {\frac{{\left( {\overline{d} - \overline{{a_{s} }} } \right) \cdot \overline{{b_{s} }} }}{2}} \right\}^{2} \cdot \sin \left( {60} \right)}}} \right]$$

### Scenarios and data

As shown above, there are two ways to maintain AED networks to provide medical aid in early OHCA treatment: Stationary AEDs illustrate a conventional approach, whereas UAS-based airborne AEDs are an innovative solution. Stationary and airborne AEDs form two overarching scenarios of AED provision, onto which three variations are applied. These scenario variations are developed to understand the effects of different inputs, to cover the uncertainty of the data and thus to secure a sound decision-making. We apply the following scenario variations:

- *Scenario variation I* shows two alternatives to provide the region of Vorpommern-Greifswald with AEDs: either covering the whole county or identifying and concentrating on historical hot spots of OHCA
- *Scenario variation II* varies the medical-induced response time, which is an AED network´s primary determinant,
- *Scenario variation III* varies unit costs of both stationary and airborne AED infrastructure.

For all scenarios modelled in this study, we assumed a brisk travelling speed for every first responder retrieving a stationary AED on foot of 0, 1 km/min. For UAS, we used an average speed of 60 km/h, which is the speed of the UAS used in the MV|LIFE|DRONE – Pilot project. This average speed takes into account the time-consuming manoeuvres for take-off and landing.

#### Scenario variation I: covered area

The variation in area coverage resulted from data provided by the Land|Rettung project. To increase the outcome of OHCAs, the project documented all OHCA cases between September 2017 and September 2019 in the district of Vorpommern-Greifswald. Using this data, hot spots with at least twelve OHCA cases within this period were identified.

**Fig. 1:**
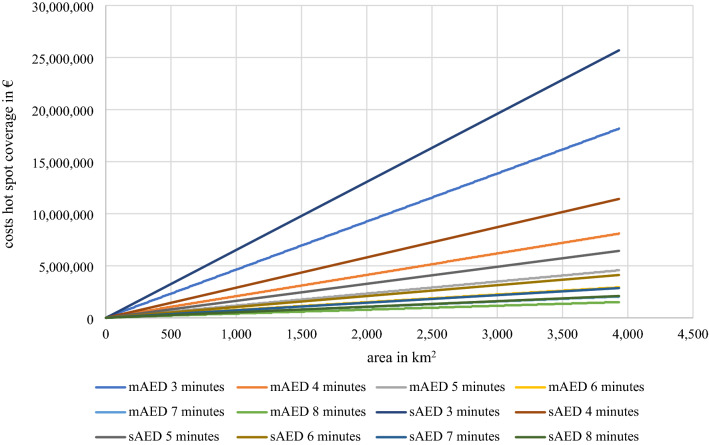
Costs for hot psot coverage. Own source

Supplementary Fig. 1 shows a heat map of all recorded OHCA in Vorpommern-Greifswald. These form the underlying base for the definition of focal points, counting 409 of the 555 emergencies between 2017 and 2019. Retrospectively, almost 75% of all OHCA cases could therefore have been covered by concentrating only on a fraction of the expanse of the exemplary region. This results in two different sized areas, constituting scenario variations 1 and 2: The entire area of Vorpommern Greifswald (3930 km^2^) and the limited area of the focal points, as shown in Fig. 1 (680.4 km^2^).

#### Scenario variation II: response time

According to the state law of Mecklenburg-Vorpommern on the provision of emergency medical services, the legal response time for EMS is 8:55 min on average [29]. However, these response times are frequently exceeded [30]. To legitimate an AED network, the delivery time of AEDs in OHCA must undercut and improve the status quo. Therefore, modelled response times vary between three and eight minutes—from very favourable to less favourable assumptions. A response time less than three minutes does not seem feasible.

#### Scenario variation III: unit costs

To cover insecurities concerning unit costs, input data are varied. For the stationary AEDs, the costs for different AED types were acquired by research. The cheapest, the most expensive, and an average price were used to depict possible cost variations. The assumed lifetime of an AED is 10 years with a linear depreciation. For the UAS costings based on the results of the MV|LIFE|DRONE project were added to the mean value of unit costs of an AED. Surmising ongoing technical development and the relatively simple UAS used in MV|LIFE|DRONE, these known costs form the bottom line of the assumed cost scale, which assumes two further and higher values.

Industry-grade octocopter-type UAS can be acquired for 127,500 €. System lifetime is assumed to be 4 years and charging cost is set at 100 €. We assume a linear depreciation of the UAS over its lifetime. Equipped with an AED of medium cost, costs per year in the base scenario are 32,248 € (Table 1).

**Table 1:** Model inputs. Own source

Scenario 1: Stationary AED Scenario 2: Aerial AED							
Scenario variation I	Scenario variation II						Scenario variation III
Covered area in km^2^	Response time *d* [min]	Radius r [km]	Catchment area A [km^2^]	Number of AEDs	*a*_*s*_; *a*_*m*_ [min]	*b*_*s*_; *b*_*m*_ [km/ min]	Costs per unit [€ p.a.]
Full area coverage [3930 km^2^]	a. 3 b. 4 c. 5 d. 6 e. 7 f. 8	${r}_{s}=\frac{\left(d-{a}_{s}\right)\bullet {b}_{s}}{2}$	$A_{s} = 3 \cdot r_{s}^{2} \cdot \sin \left( {60} \right)$	$trunc\left(\frac{x}{{A}_{s}}\right)$	1 min	0.1 km/min	a.170 € b.263 € c.405 €
Focal points [680.4 km^2^]		$r_{m} = \left( {d - a_{m} } \right) \cdot b_{m}$	$A_{m} = 3 \cdot r_{m}^{2} \cdot \sin \left( {60} \right)$	$trunc\left( {\left[ {trunc\left( {\frac{x}{{A_{m} }}} \right)} \right] \cdot 1.5} \right)$	1 min	1 km/min	a. 32,248 € b. 45,000 € c. 55,000 €

## Results

### Scenario 1: stationary AED

Table 2 shows both the total costs for the stationary AED scenario at full area coverage and focal point setting, depending on the variation of the response time and the unit costs. Increasing (decreasing) the response time enlarges (reduces) the radius of the catchment area and therefore reduces (increases) the number of AEDs required to cover the area of Vorpommern-Greifswald. Thus, the associated costs of covering a specific area decrease (response time ↑ → radius ↑ → catchment area ↑ → number of required AEDs ↓ → costs of area coverage ↓).

**Table 2:** Unit costs per scenario and variation. Own source

	Response time *d*	Scenario 1: stationary AED						Scenario 2: airborne AED					
		Radius AED [km]	Catchment per unit [km^2^]	Number AEDs needed	Costs [€ p. a.]			Radius AED [km]	Catchment per unit [km2]	Number AEDs needed	Costs [€ p.a.]		
					170 €	263 €	405 €				32,248 €	45,000 €	55,000 €
Full area coverage [3,930 km^2^]	3	0.1	0.025	151,266	25,715,220	39,782,958	61,262,730	2	10.39	569	18,349,112 €	25,605,000	31,295,000
	4	0.15	0.058	67,230	11,429,100	17,681,490	27,228,150	3	23.38	254	8,190,992 €	11,430,000	13,970,000
	5	0.2	0.103	37,817	6,428,890	9,945,871	15,315,885	4	41.56	143	4,611,464 €	6,435,000	7,865,000
	6	0.25	0.162	24,203	4,114,510	6,365,389	9,802,215	5	64.95	92	2,966,816 €	4,140,000	5,060,000
	7	0.3	0.233	16,808	2,857,360	4,420,504	6,807,240	6	93.53	65	2,096,120 €	2,925,000	3,575,000
	8	0.35	0.318	12,349	2,099,330	3,247,787	5,001,345	7	127.30	47	1,515,656 €	2,115,000	2,585,000
Hot spots [680.4 km^2^]	3	0.1	0.025	26,189	4,452,130	6,887,707	10,606,545	2	10.39	99	3,192,552 €	4,455,000	5,445,000
	4	0.15	0.058	11,640	1,978,800	3,061,320	4,714,200	3	23.38	45	1,451,160 €	2,025,000	2,475,000
	5	0.2	0.103	6,548	1,113,160	1,722,124	2,651,940	4	41.56	26	838,448 €	1,170,000	1,430,000
	6	0.25	0.162	4,191	712,470	1,102,233	1,697,355	5	64.95	17	548,216 €	765,000	935,000
	7	0.3	0.233	2,910	494,700	765,330	1,178,550	6	93.53	12	386,976 €	540,000	660,000
	8	0.35	0.318	2,138	363,460	562,294	865,890	7	127.30	9	290,232 €	405,000	495,000

To equip the full area of Vorpommern-Greifswald with stationary devices, between 12,349 and 151,266 AEDs are needed, depending on the response time as depicted in scenario variation II. If the analysis focuses on the hot spots, only between 2,138 and 26,189 AEDs are required for sufficient provision, again depending on the response time. As can be expected, increasing unit costs impact total costs in both a total coverage and focal point setting. The total costs for area coverage with stationary AEDs lie between 2,099,330 € and 61,262,730 €. When considering the hot spots, the total costs per year are set between 363,460 € and 10,606,545 € depending on the unit costs and the response time.

### Scenario 2: airborne AED

Besides the results for scenario 1, Table 2 also shows the results of response times and unit costs for area coverage with a focus on airborne AED delivery using UAS as depicted in scenario 2. Between 47 and 569 AEDs are required for a full area coverage of Vorpommern-Greifswald. Total costs range, depending on the adopted unit price, between 1,515,656 € and 31,295,000 €. Considering only focal points, between 9 and 99 AEDs are required to cover the area, causing yearly costs of between 290,232 € and 5,445,000 €.

### Cost comparison stationary and airborne AEDs

Figure 1 shows the cost for every scenario depending on the operational area based on the assumption of the lowest possible cost structures. Evidently, the application of a trunc function on fixed costs leads to an interval-fixed trend line, which, however, appears to be linear due to the figure´s high scale. Comparing the two scenarios illustrates, that the airborne UAS-based scenario shows in almost any constellation less total costs than the stationary one when comparing the same response time *d*. Airborne costs only exceed the stationary scenario when comparing the highest UAS-related cost assumption with the lowest of stationary AEDs.

**Fig. 2:**
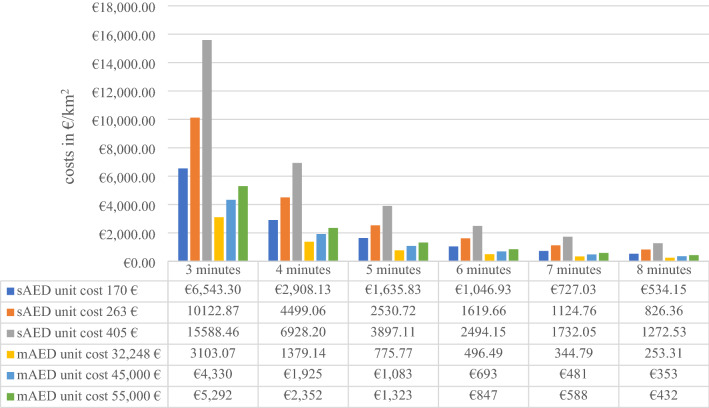
Impact of unit costs. Own source

Focusing on varying unit cost and the cost per km^2^ proves the higher cost efficiency of an airborne AED network over a stationary. Figure 2 shows the costs per km^2^, which are independent of the area coverage or focal point setting. Evidently, total costs are the highest with a response time of three minutes and fall with longer time intervals. This gives insight, as to how far the airborne response time can be reduced until the airborne costs exceed the stationary cost.

In any scenario, the cost per km^2^ operational area is lower when using an UAS. To maintain fastest availability times at the lowest assumable costs, 6543.3 € per km^2^ for stationary AEDs are more than twice as high compared to an airborne solution with 3103.07 €. If delivery times are modelled to 6 min, a significant reduction of costs to 496.49 € per km^2^ for UAS-based delivery and 1046.93 € for stationary AEDs can be noted.

### Sensitivity

By equating the cost functions and keeping all but one parameter constant, a decision maker´s indifference in choosing between the mobile or stationary alternative concerning the total costs can be determined. Variable parameters are as previously established the unit costs, the area of provision and period of help. Results are shown in Table 3.

**Table 3:** Unit costs needed to achieve indifference regarding the corresponding AED-network alternative

Base values	Unit costs stationary AEDs [€]						Unit costs aerial AEDs [€]					
	170		263		405		32,248		45,000		55,000	
Rescue time	Coverage	Hot spots	Coverage	Hot spots	Coverage	Hot spots	Coverage	Hot spots	Coverage	Hot spots	Coverage	Hot spots
3	121,30	121,90	169.27	170.11	206.89	207.91	45,193.71	44,971.01	69,917.33	69,572.80	107,667.36	107,136.82
4	121,84	124,60	170.01	173.97	207.79	212.63	44,996.46	43,973.33	69,612.17	68,029.33	107,197.44	104,760.00
5	121,94	128,05	170.16	178.68	207.98	218.39	44,957.27	42,813.85	69,55.55	66,235.54	107,104.09	101,997.69
6	122,58	130,81	171.05	182.53	209.06	223.10	44,722.93	41,910.00	69,189.01	64,837.24	106,545.82	99,844.41
7	124,71	132,98	174.02	185.57	212.70	226.80	43,959.38	41,225.00	68,007.75	63,777.50	104,726.77	98,212.50
8	122,74	135,75	171.27	189.43	209.33	231.52	44,666.60	40,384.44	69,101.85	62,477.11	106,411.60	96,210.00

For area coverage, the stationary AED´s critical unit costs range from 121.30 € to 212.70 €, to achieve indifference with the three cost levels of the mobile scenario (31,966 €, 45,000 €, 55,000 €). This means, that if the stationary AED´s cost per year ranged within this interval, the mobile alternative would no longer be more advantageous. However, based on this study´s research, that is not the case, costs per year for stationary AEDs range from 170 € upwards.

If the stationary unit costs are kept constant at the unit costs levels (170 €, 263 €, 405 €), the highest possible unit costs of an UAS-based AED can be determined, that still leaves the mobile scenario with lower or equal costs to the stationary solution. When covering the total area of Vorpommern-Greifswald, unit costs would need to range between 45,193.71 € and 107,667.36 € to achieve indifference with the total costs of the stationary scenario.

If the hot spots only are regarded, the stationary AED must have unit costs of between 121.90 € and 231.52 € to cause indifference towards the mobile scenario, depending on the response time and the related total costs. Evidently, the unit costs are below the assumed average stationary unit costs of 263 € and implicate a necessary decrease in any case. Therefore, the stationary scenario would only be advantageous compared to the mobile scenario, if unit costs were significantly lower than the average cost of a stationary AED that was used in this model.

Keeping the parameters of the stationary scenario constant, the mobile AED scenario gains indifference with unit costs between 40,384.44 € and 107,136.82 €. These costs lie mainly above the base level of mobile cost, which represents real costs determined by the MV|LIFE|DRONE project. Their top line is approximately twice as high as the highest costs level assumed in this study. Consequently, the mobile scenario is always superior to the stationary AED network, as long as the unit cost per UAS stays lower than 40,384.44 €.

## Discussion

As expected, total costs are lowest, when modelling the longest possible time interval for the AED delivery together with the lowest unit prices. However, since the provision of AEDs to OHCA is time-critical, shorter time intervals should be aimed for. This raises the question about the social willingness to pay for AED networks, to improve the outcomes of OHCA.

Given the established assumptions, this study finds that the setting of hot spots is of economic advantage, contrary to the complete coverage of a rural region with AEDs. This is substantiated in the significantly smaller area that needs to be covered. In the case of Vorpommern-Greifswald, only a sixth of the actual region would have to be covered with AEDs but would nevertheless cover about 75% of all occurring OHCA. In a two-stepped approach, covering these focal points would be the first and highly effective measure, before finding solutions to better serve the remaining expanse.

This study shows that the cost of UAS-based AED delivery is always lower than the stationary costs when comparing the same response time in corresponding scenarios. For a cost equivalence with UAS-based AEDs, the cost of stationary systems would have to be significantly reduced. It can be concluded, that even with equal system costs, mobile AED networks can achieve faster response times and therefore better medical care, than traditional stationary AED. Stationary AEDs are therefore inferior to mobile solutions.

This study is subject to a number of limitations. Firstly, the Christaller comb applied assumes that the population is evenly distributed. This theoretical assumption is not true for Vorpommern-Greifswald and not likely to be found in other regions. However, the hexagonal approach ensures that any place within it can be reached at a certain time. Therefore the Christaller comb is suitable to maintain a formally exhaustive and constant quality of health services. This limitation has been answered by the specific examination of focal points. The formal properties of the Christaller comb are opposed in this study by the specific examination of focal points to also include a focus on high-priority OHCA areas.

Furthermore, the time to retrieve the AED, response times and the respective unit cost levels are assumptions that need to be further tested and validated. This holds especially true for the conjecture that UAS are a means of transport of superior speeds compared to lay persons or first responders.

Our findings show, that UAS-supported AED delivery is superior in terms of cost efficiency than the existing stationary solutions. Due to the lack of information on clinical outcomes, we chose the access time to the AED as an effect on the cost-efficiency analysis. However, using clinical outcomes such as DALYs and QALYs would be ideal. Cost efficiency analyses and comparisons of stationary to UAS-supported AED networks should be complemented, as soon as clinical data becomes available.

UAS technology is subject to rapid innovation, enhancing their utility and operational range. Therefore, technical capabilities may impact this study´s findings. This holds true especially for the UAS´ travelling speed, which is poised to increase significantly in the future.

## Conclusion

Our aim was to evaluate the cost of alternative solutions to improve OHCA treatment. The total costs of providing AEDs in specific areas were compared by modeling two basic scenarios of a stationary AED against that of a mobile AED. Based on the exemplary rural region of Vorpommern-Greifswald, three variations were introduced, based on innovating existing EMS structures to shorten present EMS response times, unit costs and area coverage.

The calculated costs per km^2^ show that the coverage of a given area with mobile AEDs is less expensive and more effective than placing stationary ones under any simulated circumstance. Obviously, setting focal points leads to lower system costs than a full area coverage. This holds true for any region to be supplied with AEDs.

Based on these findings, supplying hotspots in the study region of Vorpommern-Greifswald with mobile AEDs with a four-minute flight radius is recommended as the most cost-efficient way to ensure a significantly shorter response time. Historically, about 75% of all past OHCA would have been covered using this strategy. Hot spot coverage would result in costs between 1,451,160 € and 2,475,000 € depending on the unit costs. As the UAS-based AED network is always more cost-efficient than the stationary, this recommendation also holds true for a full, yet more cost intensive, full area coverage.

A strong cost driver in this model is the redundant capacity of UAS, set to 50% in this study, which is deemed necessary regarding possible technical failures. Medical UAS networks could realize a lot of cost potential by utilizing aircraft with higher reliability, in that weather conditions and failure risks become much less influential. This would further the advantage of airborne AED deliveries over the stationary solution.

To achieve an applicability of the focal point solution to other regions, it is necessary to further map OHCA in order to identify high risk locations. Also, consideration should be given to how far the response time, and thus flight radii should be reduced, so that more lives can be saved in the shortest possible time without neglecting the economic aspect.

Still, the very low prospective utilization of the UAS network indicates the possibility of integrating possible other use cases into the network. Those could be the transport of other medical goods, such as laboratory samples or the use for situations assessments in crisis management. The availability of ED delivery to OHCA could be ensured by always carrying during one on non-related transports.

## Supplementary Information

Below is the link to the electronic supplementary material.

Supplementary file1 (DOCX 410 KB)

Supplementary file2 (DOCX 14 KB)

## Abbreviations

AED: Automatic external defibrillator
CFR: Community first responder
CPR: Cardiopulmonary resuscitation
EMS: Emergency medical services
OHCA: Out-of-hospital cardiac arrest
UAS: Unmanned aerial systems
