# Supplementary figures and images for: Can drones save lives and money? An economic evaluation of airborne delivery of automated external defibrillators
Source: Eur J Health Econ. 2022 Oct 30;24(7):1141–50. doi: 10.1007/s10198-022-01531-0 (PMC10406671; doi:10.1007/s10198-022-01531-0)

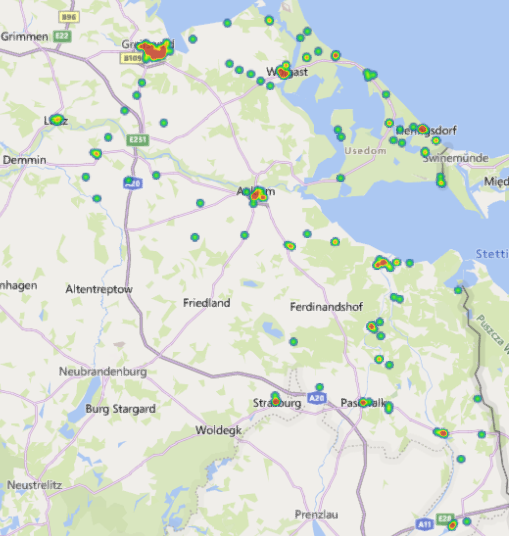


Figure 1: Locations of registered OHCA from 2017-2019. Source: Project Land|Rettung, own.

Supplement: Supplementary file 1 — Supplementary file1 (DOCX 410 KB) [file 10198_2022_1531_MOESM1_ESM.docx]
