# Supplementary material for: Can drones save lives and money? An economic evaluation of airborne delivery of automated external defibrillators
Source: Eur J Health Econ. 2022 Oct 30;24(7):1141–50. doi: 10.1007/s10198-022-01531-0 (PMC10406671; doi:10.1007/s10198-022-01531-0)
Supplement: Supplementary file 2 — Supplementary file2 (DOCX 14 KB) [file 10198_2022_1531_MOESM2_ESM.docx]

| Location | Area [km^2^] | OHCA cases |
| --- | --- | --- |
| Ahlbeck | 18,7 | 18 |
| Anklam | 56,6 | 41 |
| Ferdinandshof, Lübs | 77,4 | 16 |
| Greifswald | 50,7 | 97 |
| Heringsdorf | 37,7 | 14 |
| Karlshagen | 5,1 | 18 |
| Koserow, Zempin | 9,4 | 20 |
| Löcknitz | 22,6 | 12 |
| Pasewalk | 55 | 37 |
| Strasburg | 86,8 | 12 |
| Torgelow | 72,2 | 26 |
| Ueckermünde | 84,6 | 26 |
| Wolgast | 61,5 | 39 |
| Wusterhusen, Lubmin | 33 | 18 |
| Zinnowitz | 9,1 | 15 |
| total | 680,4 | 409 |

Supplementary table 1: Locations of OHCA hot spots. Own source.
